# Synthesis, Antimicrobial and Hypoglycemic Activities of Novel *N*-(1-Adamantyl)carbothioamide Derivatives

**DOI:** 10.3390/molecules20058125

**Published:** 2015-05-06

**Authors:** Ebtehal S. Al-Abdullah, Hanaa M. Al-Tuwaijri, Hanan M. Hassan, Monirah A. Al-Alshaikh, Elsayed E. Habib, Ali A. El-Emam

**Affiliations:** 1 Department of Pharmaceutical Chemistry, College of Pharmacy, King Saud University, Riyadh 11451, Saudi Arabia; E-Mails: ealabdullah@ksu.edu.sa (E.S.A.-A.); haltuwajri@ksu.edu.sa (H.M.A.-T.); 2 Department of Pharmaceutical Sciences, College of Pharmacy, Princess Nourah Bint Abdulrahman University, Riyadh 11671, Saudi Arabia; E-Mail: hananhafila@hotmail.com; 3 Department of Chemistry, College of Sciences, King Saud University, Riyadh 11451, Saudi Arabia; E-Mail: mshaikh@ksu.edu.sa; 4 Department of Microbiology, Faculty of Pharmacy, University of Mansoura, Mansoura 35516, Egypt; E-Mail: sayedhabib@hotmail.co.jp; 5 Department of Pharmaceutics and Pharmaceutical Technology (Microbiology), College of Pharmacy, Taibah University, Almadinah Almunawwarah 11344, Saudi Arabia

**Keywords:** adamantane derivatives, carbothioamides, antimicrobial activity, hypoglycemic activity

## Abstract

The reaction of 1-adamantyl isothiocyanate **4** with the various cyclic secondary amines yielded the corresponding *N*-(1-adamantyl)carbothioamides **5a**–**e**, **6**, **7**, **8a**–**c** and **9**. Similarly, the reaction of **4** with piperazine and *trans*-2,5-dimethylpiperazine in 2:1 molar ratio yielded the corresponding *N*,*N'*-bis(1-adamantyl)piperazine-1,4-dicarbothioamides **10a** and **10b**, respectively. The reaction of *N*-(1-adamantyl)-4-ethoxycarbonylpiperidine-1-carbothioamide **8c** with excess hydrazine hydrate yielded the target carbohydrazide **11**, in addition to 4-(1-adamantyl)thiosemicarbazide **12** as a minor product. The reaction of the carbohydrazide **11** with methyl or phenyl isothiocyanate followed by heating in aqueous sodium hydroxide yielded the 1,2,4-triazole analogues **14a** and **14b**. The reaction of the carbohydrazide **11** with various aromatic aldehydes yielded the corresponding *N'*-arylideneamino derivatives **15a**–**g**. The compounds **5a**–**e**, **6**, **7**, **8a**–**c**, **9**, **10a**, **10b**, **14a**, **14b** and **15a**–**g** were tested for *in vitro* antimicrobial activity against certain strains of pathogenic Gram-positive and Gram-negative bacteria and the yeast-like fungus *Candida albicans*. The compounds **5c**, **5d**, **5e**, **6**, **7**, **10a**, **10b**, **15a**, **15f** and **15g** showed potent antibacterial activity against one or more of the tested microorganisms. The oral hypoglycemic activity of compounds **5c**, **6**, **8b**, **9**, **14a** and **15b** was determined in streptozotocin (STZ)-induced diabetic rats. Compound **5c** produced significant reduction of serum glucose levels, compared to gliclazide.

## 1. Introduction

Derivatives of adamantane have long been known for their multifarious pharmacological activities. The incorporation of an adamantyl moiety into several molecules results in compounds with relatively high lipophilicity, which in turn can modify the biological availability of these molecules. The adamantyl-bearing compound are more lipophilic than their des-adamantyl analogues. Beyond increasing the partition coefficient, the adamantyl group positively modulates the therapeutic index of many experimental compounds through a variety of mechanisms [1,2]. After the discovery of amantadine in 1960 as potent antiviral drug for the treatment of Influenza A infection [3,4,5] and as antiparkinsonian drug [6,7], adamantane derivatives attracted the attention of several scientists as potential chemotherapeutic agents. Further studies based on amantadine resulted in the discovery of more potent antiviral drugs as Rimantadine [8] and Tromantadine [9]. Several adamantane derivatives were also proved to possess marked inhibitory activity against human immunodeficiency viruses (HIV) [10,11,12,13]. 6-[3-(1-Adamantyl)-4-hydroxyphenyl]-2-naphthalene carboxylic acid (CD437), a synthetic retinoid derivative, was developed as a potent inducer of apoptosis in human head and neck squamous cell carcinoma [14]. Several adamantane derivatives were recognized as potent bactericidal and fungicidal agents [15,16,17,18,19,20,21,22,23,24,25]. In addition, the adamantane-based drugs, Vildagliptin [26] and Saxagliptin [27] are members of a new class of oral hypoglycemic agents known as dipeptidyl peptidase IV (DPP-IV) inhibitors, which were approved for the treatment of type 2 diabetes. Adamantane derivatives constitutes the major class of 11β-hydroxysteroid dehydrogenase type 1 (11β-HSD1) inhibitor, which are considered important therapy for controlling non-insulin-dependent diabetes, hyperglycemia, obesity, insulin resistance, hyperlipidemia, hypertension and other symptoms associated with excessive body cortisol [28,29,30]. Moreover, anti-inflammatory activity was reported to several adamantane-containing molecules [19,20,21,22,31,32,33,22,31]. In view of the reported chemotherapeutic and hypoglycemic activities of the *N*-(1- and 2-adamantyl)carboxamides [29,34,35,36] and the diverse biological activities of several *N*-(substituted)carbothioamides [37,38], it was of interest to synthesize series of *N*-(1-adamanty)carbothioamide derivatives, structurally-related to the previously reported adamantane derivatives, for evaluation as antimicrobial and hypoglycemic agents.

## 2. Results and Discussion

### 2.1. Chemical Synthesis

**Scheme 1:**
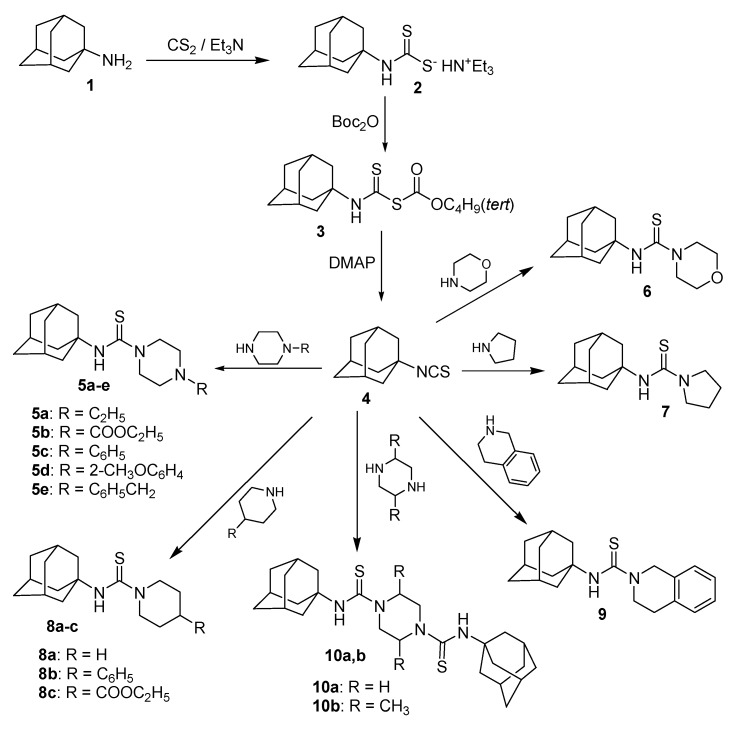
Synthesis of the target *N*-(1-adamanty)carbothioamides **5a**–**e**, **6**, **7**, **8a**–**c**, **9**, **10a** and **10b**.

1-Adamantyl isothiocyanate **4**, required as starting material, was prepared in good yield via modification of the previously described methods [39,40]. Thus, 1-adamantylamine **1** was reacted with carbon disulfide and trimethylamine, in ethanol, to yield the dithiocarbamate salt **2**, followed by addition of di-*tert*-butyl dicarbonate (Boc_2_O) to yield the intermediate **3**, which was converted to the target product **4** via stirring with catalytic amount of 4-dimethylaminopyridine (DMAP). 1-Adamantyl isothiocyanate **4** was reacted with the cyclic secondary amines namely, 1-substituted piperazines, morpholine, pyrrolidine, 4-substituted piperdines and 1,2,3,4-tetrahydroisoquinoline, in boiling ethanol, to yield the corresponding *N*-(1-adamantyl)carbothioamides **5a**–**e**, **6**, **7**, **8a**–**c** and **9**, respectively. The reaction was found to proceed smoothly and the products were precipitated from the reaction mixture in good yields after two hours. 1-Adamantyl isothiocyanate **4** was similarly reacted with piperazine and *trans*-2,5-dimethylpiperazine in 2:1 molar ratio to yield the corresponding *N*,*N'*-bis(1-adamantyl)piperazine-1,4-dicarbothioamide derivatives **10a** and **10b** in high yields (Scheme 1, Table 1). The structures of compounds **5a**–**e**, **6**, **7**, **8a**–**c**, **9**, **10a** and **10b** were confirmed by elemental analyses, in addition to the ^1^H-NMR, ^13^C-NMR, and ESI-MS mass spectral data which were in full agreement with their structures, in addition to the X-ray spectrum of compound **9** [41].

**Table 1:** Crystallization solvents, melting points, yield percentages, molecular formulae and molecular weights of compounds **5a**–**e**, **6**, **7**, **8a**–**c**, **9**, **10a**, **10b**, **14a**, **14b** and **15a**–**g**.

Comp. No.	R	Cryst. Solv.	Mp (°C)	Yield (%)	Molecular Formula (Mol. Wt.)
**5a**	C_2_H_5_	EtOH	150–152	77	C_17_H_29_N_3_S (307.50)
**5b**	COOC_2_H_5_	EtOH	121–123	68	C_18_H_29_N_3_O_2_S (351.51)
**5c**	C_6_H_5_	EtOH	174–176	91	C_21_H_29_N_3_S (355.54)
**5d**	2-CH_3_OC_6_H_4_	EtOH	137–139	95	C_22_H_31_N_3_OS (385.57)
**5e**	C_6_H_5_CH_2_	EtOH	146–148	95	C_22_H_31_N_3_S (369.57)
**6**	-	EtOH	139–141	88	C_15_H_24_N_2_OS (280.43)
**7**	-	EtOH	175–177	74	C_15_H_24_N_2_S (264.43)
**8a**	H	EtOH	145–147	78	C_16_H_26_N_2_S (278.46)
**8b**	C_6_H_5_	EtOH	137–139	90	C_22_H_30_N_2_S (354.55)
**8c**	COOC_2_H_5_	EtOH	166–168	78	C_19_H_30_N_2_O_2_S (350.52)
**9**	-	EtOH	147–149	88	C_20_H_26_N_2_S (326.5)
**10a**	H	DMF	227–229	89	C_26_H_40_N_4_S_2_ (472.75)
**10b**	CH_3_	DMF	232–234	92	C_28_H_44_N_4_S_2_ (500.81)
**14a**	CH_3_	EtOH	200–202	38	C_19_H_29_N_5_S_2_ (391.6)
**14b**	C_6_H_5_	DMF	>300	44	C_24_H_31_N_5_S_2_ (453.67)
**15a**	2-OH	EtOH	196–198	65	C_24_H_32_N_4_O_2_S (440.6)
**15b**	3,4-Cl_2_	EtOH/CHCl_3_	237–237	61	C_24_H_30_Cl_2_N_4_OS (493.49)
**15c**	2,6-Cl_2_	EtOH	199–201	54	C_24_H_30_Cl_2_N_4_OS (493.49)
**15d**	3,4-(OCH_3_)_2_	EtOH	154–156	48	C_26_H_36_N_4_O_3_S (484.65)
**15e**	3,4,5-(OCH_3_)_3_	EtOH	128–130	43	C_27_H_38_N_4_O_4_S (514.68)
**15f**	2-OH-5-OCH_3_	EtOH/CHCl_3_	170–172	44	C_25_H_34_N_4_O_3_S (470.63)
**15g**	3-OC_2_H_5_-4-OH	EtOH/CHCl_3_	194–196	58	C_26_H_36_N_4_O_3_S (484.65)

*N*-(1-Adamantyl)-4-ethoxycarbonylpiperidine-1-carbothioamide **8c** was reacted with excess hydrazine hydrate, in ethanol, at reflux temperature, to get the target carbohydrazide derivative **11**. On monitoring the reaction with thin layer chromatography (TLC), it was observed that the target product was formed after few minutes in addition to a minor product which was further identified as 4-(1-adamantyl)thiosemicarbazide **12**. It was also observed that prolongation of the reaction time results in higher ratios of the side product **12**. The reaction time was optimized at 20 min to yield 72% of **11** and 18% of **12**. The formation of the side product could be explained as a result of hydrazinolysis of the thiocarboxamide function of the major product **11**. The structure of the side product **12** was assigned based on the ^1^H-NMR and ^13^C-NMR data, in addition to ESI-MS mass spectra and elemental analyses.

The reaction of the carbohydrazide **11** with equimolar amount of methyl or phenyl isothiocyanate, in ethanol for 6 h yielded the intermediate 1,4-disubstituted-3-thiosemicarbazides **13a** or **13b**. Dehydrative cyclization of compounds **13a** and **13b** was achieved by heating in 10% aqueous sodium hydroxide solution for 2 h, followed by acidification with hydrochloric acid to yield the target 1,2,4-triazole derivatives **14a** and **14b** in 38% and 44% overall yields, respectively. Attempted reaction of the carbohydrazide **11** with various aromatic aldehydes via prolonged heating in ethanol yielded fair yields of the corresponding arylideneamino derivatives. On the other hand, carrying out the reaction in the higher boiling solvent *N*,*N*-dimethylformamide (DMF) greatly improved the yield. Thus, the reaction of the carbohydrazide **11** with certain aromatic aldehydes via heating in DMF for two hours yielded the target compounds **15a**–**g** in relatively higher yields (43%–71%).

**Scheme 2:**
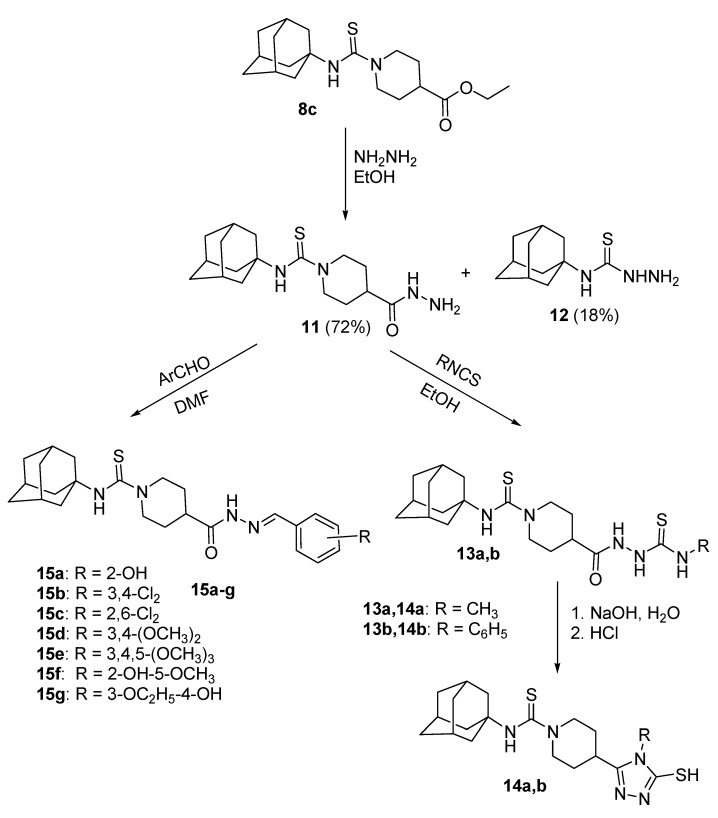
Synthesis of the target *N*-(1-adamanty)carbothioamides **14a**,**b** and **15a**–**h**.

The structures of compounds **11**, **12**, **14a**, **14b** and **15a**–**g** (Scheme 2, Table 1) were confirmed by elemental analyses, in addition to the ^1^H-NMR, ^13^C-NMR, and ESI-MS mass spectral data, which were in full agreement with their structures.

### 2.2. In Vitro Antimicrobial Activity

The synthesized compounds **5a**–**e**, **6**, **7**, **8a**–**c**, **9**, **10a**, **10b**, **14a**, **14b** and **15a**–**g** were tested for *in vitro* inhibitory activity against the standard pathogenic strains of the Institute of fermentation of Osaka (IFO) namely; *Staphylococcus aureus* IFO 3060, *Bacillus subtilis* IFO 3007, *Micrococcus luteus* IFO 3232 (Gram-positive bacteria), *Escherichia coli* IFO 3301, *Pseudomonas aeuroginosa* IFO 3448 (Gram-negative bacteria), and the yeast-like pathogenic fungus *Candida albicans* IFO 0583. The primary screening was carried out using the agar disc-diffusion method using Müller-Hinton agar medium [42]. The results of the preliminary antimicrobial screening of the synthesized compounds (200 μg/disc), the antibacterial antibiotics Ampicillin trihydrate, Gentamicin (100 μg/disc) and the potent antifungal drug Clotrimazole (100 μg/disc) and the calculated log *P* values (Clog *P*) of the tested compounds (calculated using the CS ChemOffice Ultra version 8.0, CambridgeSoft, Cambridge, MA, USA) are presented in Table 2.

**Table 2:** Antimicrobial activity of compounds **5a**–**e**, **6**, **7**, **8a**–**c**, **9**, **10a**, **10b**, **14a**, **14b** and **15a**–**g** (200 μg/8 mm disc), the broad spectrum antibacterial drugs Gentamicin (100 μg/8 mm disc), Ampicillin (100 μg/8 mm disc) and the antifungal drug Clotrimazole (100 μg/8 mm disc) against *Staphylococcus aureus* IFO 3060 (*SA*), *Bacillus subtilis* IFO 3007 (*BS*), *Micrococcus luteus* IFO 3232 (ML), *Escherichia coli* IFO 3301 (*EC*), *Pseudomonas aeuroginosa* IFO 3448 (*PA*), and *Candida albicans* IFO 0583 (*CA*).

Comp. No.	Clog *P*	Diameter of Growth Inhibition Zone (mm) ^a^					
		*SA*	*BS*	*ML*	*EC*	*PA*	*CA*
**5a**	4.32	-	-	-	-	-	-
**5b**	4.32	-	-	-	-	-	-
**5c**	4.77	20 (8) ^b^	24 (2) ^b^	16	15	14	-
**5d**	4.79	19 (4) ^b^	22 (4) ^b^	17	12	-	-
**5e**	5.52	15	19 (8) ^b^	12	15	12	-
**6**	3.23	22 (2) ^b^	20 (1) ^b^	19 (4) ^b^	15	12	-
**7**	4.06	18 (8) ^b^	20 (2) ^b^	18 (4) ^b^	15	13	-
**8a**	4.62	12	14	12	-	-	-
**8b**	6.03	16	16	14	-	-	-
**8c**	4.01	12	13	-	-	-	-
**9**	5.48	-	-	-	-	-	-
**10a**	6.86	25 (32) ^b^	28 (16) ^b^	20 (32) ^b^	18	16	-
**10b**	7.90	26 (32) ^b^	24 (8) ^b^	23 (32) ^b^	19 (64) ^b^	12	-
**14a**	3.40	-	-	-	-	-	-
**14b**	5.29	-	-	-	-	-	-
**15a**	5.07	18 (16) ^b^	22 (2) ^b^	15	12	-	-
**15b**	5.77	12	12	-	-	-	-
**15c**	4.70	14	17	12	-	-	-
**15d**	4.41	-	-	-	-	-	-
**15e**	4.03	-	-	-	-	-	-
**15f**	5.14	22 (8) ^b^	27 (1) ^b^	19 (8) ^b^	13	11	11
**15g**	4.79	19 (8) ^b^	25 (2) ^b^	18 (8) ^b^	12	12	13
**Gentamicin**		26 (2) ^b^	25 (2) ^b^	18 (2) ^b^	20 (0.5) ^b^	19 (1) ^b^	NT
**Ampicillin**		23 (2) ^b^	21 (0.5) ^b^	19 (2) ^b^	17 (2) ^b^	16 (2) ^b^	NT
**Clotrimazole**		NT	NT	NT	NT	NT	21 (2) ^b^

^a^ (-): Inactive (inhibition zone < 10 mm); (NT): Not tested; ^b^ The figures shown in parentheses represent the MIC values (μg/mL).

The antimicrobial activity results revealed that the tested compounds exhibited various degrees of inhibition against the tested microorganisms. Potent antibacterial activity was displayed by the compounds **5c**–**e**, **6**, **7**, **10a**, **10b**, **15a**, **15f** and **15g** which produced growth inhibition zones ≥ 18 mm against one or more of the tested bacteria. In addition, the derivatives **8a**, **8b** and **15c** showed moderate activity (growth inhibition zones 14–17 mm), the derivatives **8c** and **15b** produced weak activity (growth inhibition zones 10–13 mm) and the derivatives **5a**, **5b**, **9**, **14a**, **14b**, **15d** and **15e** were practically inactive (growth inhibition zones < 10 mm) against the tested microorganisms.

The Gram-positive bacteria *Bacillus subtilis* and *Staphylococcus aureus* and to a lesser extent *Micrococcus luteus* are considered the most sensitive among the tested microorganisms. Meanwhile, the activity against the tested Gram-negative bacteria was generally lower than that of the Gram-positive bacteria, only compound **10a** and **10b** were found strongly active against *Escherichia coli* and moderately or weakly active against *Pseudomonas aeuroginosa*. The inhibitory activity of the compounds against *Candida albicans* was rather lower than their antibacterial activity, only compounds **15f** and **15g** displayed marginal activity compared to Clotrimazole. In addition, the antimicrobial activity of the compounds were not correlated to their lipophilicity.

The *N*-(1-adamantyl)carbothioamides **5c**–**e**, **6**, **7** and **8a**–**c** showed marked activity against the tested Gram-positive bacteria and weak to moderate activity against the tested Gram-negative bacteria, in addition to the absence of antifungal activity. The antibacterial activity was dependent on the nature of the precursor cyclic secondary amine. Among the piperazine derivatives **5a**–**e**, the 4-aryl and benzyl derivatives **5c**, **5d** and **5e** were highly active and the ethyl and the ethoxycarbonyl derivatives **5a** and **5b** were inactive. The morpholine and pyrrolidine derivatives **6** and **7** were highly active against the Gram-positive bacteria and retained moderate activity against *Escherichia coli* and weak activity against *Pseudomonas aeuroginosa*. The antibacterial activity of the piperidine derivatives **8a**–**c** was lower than their morpholine and pyrrolidine analogues with moderate to weak activity against the tested Gram-positive bacteria. The tetrahydroisoquinoline derivative **9** totally lacked antimicrobial activity.

The *N*,*N'*-bis(1-adamantyl)piperazine-1,4-dicarbothioamides **10a** and **10b** exhibited potent broad-spectrum antibacterial activity against the tested Gram-positive bacteria and *Escherichia coli* in addition moderate activity and weak activity against *Pseudomonas aeuroginosa*. The *N*-(1-adamantyl)piperidine-4-(5-mercapto-4-phenyl-1,2,4-triazol-3-yl)-1-carbothioamides **14a** and **14b** totally lacked antimicrobial activity. Concerning the antimicrobial activity of the *N'*-(arylidene)piperidine-4-carbohydrazides **15a**–**g**, it was observed that the activity mainly dependent on the arylidene moiety. The phenolic derivatives **15a**, **15f** and **15g** displayed potent antibacterial activity against the tested Gram-positive bacteria and endowed marginal activity against *Candida albicans*.

The minimal inhibitory concentrations (MIC) [43] for the most active compounds **5c**, **5d**, **5e**, **6**, **7**, **10a**, **10b**, **15a**, **15f** and **15g** which are shown in Table 2, were in accordance with the results obtained in the primary screening. Despite the potent broad-spectrum antibacterial activity of compounds **10a** and **10b**, the MIC values were higher than expected. The high MIC values of compounds **10a** and **10b** may be attributed high lipophilicity and the poor water solubility in the aqueous Müller-Hinton Broth and Sabouraud Liquid Medium.

### 2.3. In Vivo Hypoglycemic Activity

The oral hypoglycemic activity of compounds **5c**, **6**, **8b**, **9**, **14a** and **15b** was determined in streptozotocin (STZ)-induced diabetic rats. The compounds were tested at 10 and 20 mg/kg dose levels. The diabetogenic effect of STZ is the direct result of irreversible damage to the pancreatic beta cells, resulting in degranulation and loss of insulin secretion [44,45].

The results of oral hypoglycemic activity compounds **5c**, **6**, **8b**, **9**, **14a** and **15b** (10 and 20 mg/kg) and the potent hypglycemic drug gliclazide in STZ-induced diabetic rats (10 mg/kg) are listed in Table 3. The highest activity was shown by compound **5c**, which produced significant strong dose-independent reduction of serum glucose levels in STZ-induced diabetic rats, compared to gliclazide at 10 mg/kg dose level (Potency ratio 92.48%). Compound **6** displayed good hypoglycemic at 20 mg/kg dose level and weak activity at 10 mg/kg dose level.

**Table 3:** Oral hypoglycemic activity of compounds **5c**, **6**, **8b**, **9**, **14a**, **15b** (10 and 20 mg/kg) and gliclazide (10 mg/kg) in STZ-induced diabetic rats.

Treatment	Results		
	C_0_ (mg/dL) ^a^	C_24_ (mg/dL) ^a^	% Glucose Reduction ^b^
**Group 1 ^c^**	302.6 ± 11.64	287.2 ± 16.85	5.09%
**Group 2 ^d^**	295.4 ± 17.52	183.0 ± 13.38 *	38.05%
**5c** (10 mg/kg)	291.6 ± 15.23	189.0 ± 22.16 *	35.19% (92.48)
**5c** (20 mg/kg)	319.6 ± 7.85	207.0 ± 13. 84 *	35.23% (46.30)
**6** (10 mg/kg)	283.8 ± 11.16	263.8 ± 16.66	7.05% (18.52)
**6** (20 mg/kg)	296.6 ± 12.92	196.0 ± 9.67 *	33.92% (44.58)
**8b** (10 mg/kg)	264.2 ± 5.49	257.4 ± 9.45	2.57% (6.75)
**8b** (20 mg/kg)	303.3 ± 16.35	291.2 ± 8.18	4.05% (5.32)
**9** (10 mg/kg)	282.8 ± 13.90	275.8 ± 15.52	2.48% (6.52)
**9** (20 mg/kg)	276.2 ± 14.17	264.6 ± 7.35	4.20% (5.52)
**14a** (10 mg/kg)	274.8 ± 14.59	277.4 ± 7.19	0.95% (2.50)
**14a** (20 mg/kg)	286.4 ± 24.56	271.4 ± 21.85	5.24% (6.89)
**15b** (10 mg/kg)	282.0 ± 14.21	287.8 ± 13.37	−2.06%
**15b** (20 mg/kg)	293.2 ± 15.66	286.8 ± 17.16	2.18% (2.87)

^a^ Results are expressed as mean ± S.E.M. (n = 5); ^b^ The figures shown in parentheses are the relative potency compared with glicalzide; ^c^ Treated with a single oral dose of 0.5% (w/v) aqueous CMC solution (5 mL/kg); ^d^ Treated with 10 mg/kg gliclazide in 0.5% (w/v) aqueous CMC; * Significant difference at *p* < 0.01 compared with the corresponding control.

The hypoglycemic activity of the tested *N*-adamantyl carbothioamides **5c**, **6**, **8b**, **9**, **14a** and **15c** greatly influenced by the nature of the carbothioamide moiety. The piperazine and morpholine carbothioamides **5c** and **6** retained good potency, while the corresponding piperidine and tetrahydroisoquinoline analogs **8b**, **9**, **14a** and **15c** were almost inactive.

### 2.4. Oral Acute Toxicity Testing of Compound **5c**

The method of Litchfield and Wilcoxon was adopted for measuring the acute oral toxicity of compound **5c** which possessed the highest hypoglycemic activity [46]. The oral LD_50_ of compound **5c** in normal albino mice was found to be 298 ± 15.50 mg/kg. The oral LD_50_ of gliclazide was reported to be >3000 mg/kg in mice [47]. Although the oral acute toxicity of compound **5c** is higher than that of gliclazide, the compound induces its hypoglycemic activity at safe doses.

## 3. Experimental Section

### 3.1. General

Melting points (°C) were measured in open glass capillaries using a Branstead 9100 Electrothermal melting point apparatus and are uncorrected. NMR spectra were obtained on a Bruker AC 500 Ultra Shield NMR spectrometer (Fällanden, Switzerland) operating at 500.13 MHz for ^1^H and 125.76 MHz for ^13^C, the chemical shifts are expressed in δ (ppm) downfield from tetramethylsilane (TMS) as internal standard; coupling constants (*J*) are expressed in Hz. Electrospray ionization mass spectra (ESI-MS) were recorded on an Agilent 6410 Triple Quad tandem mass spectrometer at 4.0 and 3.5 kV for positive and negative ions, respectively. Elemental analyses (C, H, N & S) were in agreement with the proposed structures within ±0.4% of the theoretical values. Monitoring the reactions and checking the purity of the final products were carried out by thin layer chromatography (TLC) using silica gel precoated aluminum sheets (60 F_254_, Merck) and visualization with ultraviolet light (UV) at 365 and 254 nm. The bacterial strains and *Candida albicans* fungus were obtained from the Institute of Fermentation of Osaka (IFO), Osaka, Japan. The reference drugs Ampicillin trihydrate (CAS 7177-48-2), Gentamicin sulfate (CAS 1405-41-0), Clotrimazole (CAS 23593-75-1) and Gliclazide (CAS 21187-98-4) were purchased from Sigma-Aldrich Chemie GmbH, Taufkirchen, Germany. The Sprauge-Dawley rats and the normal albino mice were purchased from local animal house (Abu-Rawash, Giza, Egypt). The animal experiments for the determination of the hypoglycemic activity and acute toxicity were carried out in agreement with the pertinent legal and ethical standards of the international guidelines.

### 3.2. Synthesis of N-(1-Adamantyl)-4-substituted piperazine-1-carbothioamides **5a**–**e**, N-(1-Adamantyl)morpholine-4-carbothioamide **6**, N-(1-Adamantyl)pyrrolidine-1-carbothioamide **7**, N-(1-Adamantyl)-4-substituted piperidine-1-carbothioamides **8a**–**c** and N-(1-Adamantyl)-1,2,3,4-tetrahydroisoquinoline-2-carbothioamide **9**

A mixture of 1-adamantyl isothiocyanate **4** (387 mg, 2 mmol) and 2.0 mmol of the appropriate cyclic secondary amine (1-substituted piperazines, morpholine, pyrrolidine, piperidine, 4-phenylpiperidine, ethyl isonipecotate or 1,2,3,4-tetrahydroisoquinoline), in ethanol (15 mL), was heated under reflux for 2 h. On cooling, the precipitated crude product were filtered, washed with cold ethanol, dried, and crystallized from ethanol.

**5a**: ^1^H-NMR (DMSO-*d_6_*): δ 1.0 (t, 3H, CH_2_C***H***_3_, *J* = 7.0 Hz), 1.62 (s, 6H, Adamantane-H), 1.97–2.03 (m, 3H, Adamantane-H), 2.24 (s, 6H, Adamantane-H), 2.32 (s, 4H, Piperazine-H), 3.34 (q, 2H, C***H***_2_CH_3_, *J* = 7.0 Hz), 3.68 (s, 4H, Piperazine-H), 6.52 (s, 1H, NH). ^13^C-NMR: δ 11.90 (***C***H_3_CH_2_), 29.06, 39.15, 39.97, 40.90 (Adamantane-C), 47.18 (***C***H_2_CH_3_), 51.35, 52.14 (Piperazine-C), 180.21 (C=S). ESI-MS, *m/z*: 308.3 (M+H)^+^.

**5b**: ^1^H-NMR (DMSO-*d_6_*): δ 1.18 (t, 3H, CH_2_C***H***_3_, *J* = 7.0 Hz), 1.58–1.62 (m, 6H, Adamantane-H), 1.78 (s, 3H, Adamantane-H), 2.25 (s, 6H, Adamantane-H), 3.34–3.39 (m, 4H, Piperazine-H), 3.73 (s, 4H, Piperazine-H), 4.04 (q, 2H, C***H***_2_CH_3_, *J* = 7.0 Hz), 6.57 (s, 1H, NH). ^13^C-NMR: δ 14.54 (***C***H_3_CH_2_), 29.09, 36.08, 39.06, 40.06 (Adamantane-C), 46.72, 53.84 (Piperazine-C), 60.69 (***C***H_2_CH_3_), 154.63 (C=O), 180.53 (C=S). ESI-MS, *m/z*: 352.4 (M+H)^+^.

**5c**: ^1^H-NMR (DMSO-*d_6_*): δ 1.63 (s, 6H, Adamantane-H), 1.96–2.05 (s, 3H, Adamantane-H), 2.27 (s, 6H, Adamantane-H), 3.16–3.20 (m, 4H, Piperazine-H), 3.84–3.88 (m, 4H, Piperazine-H), 6.64 (s, 1H, NH), 6.77–6.80 (m, 1H, Ar-H), 6.92–6.94 (m, 2H, Ar-H), 7.21–7.24 (m, 2H, Ar-H). ^13^C-NMR: δ 29.08, 36.08, 40.90, 46.96 (Adamantane-C), 47.66, 53.80 (Piperazine-C), 115.15, 118.82, 128.95, 150.48 (Ar-C), 180.41 (C=S). ESI-MS, *m/z*: 356.4 (M+H)^+^.

**5d**: ^1^H-NMR (CDCl_3_): δ 1.58–1.66 (m, 6H, Adamantane-H), 1.90–2.05 (s, 3H, Adamantane-H), 2.24 (s, 6H, Adamantane-H), 3.03 (s, 4H, Piperazine-H), 3.80 (s, 3H, OCH_3_), 3.84–3.88 (m, 4H, Piperazine-H), 5.23 (s, 1H, NH), 6.81–6.86 (m, 3H, Ar-H), 6.95–6.96 (m, 1H, Ar-H). ^13^C-NMR: δ 29.76, 35.54, 36.44, 41.92 (Adamantane-C), 47.29, 53.18 (Piperazine-C), 55.44 (OCH_3_), 111.38, 118.36, 121.10, 123.51, 140.48, 152.24 (Ar-C), 180.49 (C=S). ESI-MS, *m/z*: 386.4 (M+H)^+^.

**5e**: ^1^H-NMR (DMSO-*d_6_*): δ 1.62 (s, 6H, Adamantane-H), 1.97–2.03 (s, 3H, Adamantane-H), 2.24 (s, 6H, Adamantane-H), 2.34 (s, 4H, Piperazine-H), 3.49 (s, 2H, CH_2_), 3.70 (s, 4H, Piperazine-H), 6.52 (s, 1H, NH), 7.27–7.34 (m, 5H, Ar-H). ^13^C-NMR: δ 29.06, 36.07, 39.05, 40.91 (Adamantane-C), 47.21, 52.27 (Piperazine-C), 61.68 (CH_2_), 126.98, 128.16, 128.92, 137.72 (Ar-C), 180.19 (C=S). ESI-MS, *m/z*: 370.4 (M+H)^+^.

**6**: ^1^H-NMR (DMSO-*d_6_*): δ 1.63 (s, 6H, Adamantane-H), 1.97–2.05 (s, 3H, Adamantane-H), 2.26 (s, 6H, Adamantane-H), 3.57 (s, 4H, Morpholine-H), 3.67 (s, 4H, Morpholine-H), 6.57 (s, 1H, NH). ^13^C-NMR: δ 29.08, 36.08, 40.85, 47.69 (Adamantane-C), 53.78, 65.79 (Morpholine-C), 180.87 (C=S). ESI-MS, *m/z*: 281.3 (M+H)^+^.

**7**: ^1^H-NMR (CDCl_3_): δ 1.68–1.73 (m, 7H, 3 Adamantane-H & 4 Pyrrolidine-H), 1.98–2.02 (m, 3H, Adamantane-H), 2.09–2.12 (s, 3H, Adamantane-H), 2.22–2.23 (m, 6H, Adamantane-H), 3.55–3.57 (m, 4H, Pyrrolidine-H), 4.98 (s, 1H, NH). ^13^C-NMR: δ 25.52, 54.50 (Pyrrolidine-C), 29.83, 36.37, 41.99, 42.14 (Adamantane-C), 176.68 (C=S). ESI-MS, *m/z*: 265.3 (M+H)^+^.

**8a**: ^1^H-NMR (CDCl_3_): δ 1.52–1.66 (m, 12H, Piperidine & Adamantane-H), 2.04 (s, 3H, Adamantane-H), 2.22–2.23 (m, 6H, Adamantane-H), 3.65 (t, 4H, Piperidine-H, *J* = 5.5 Hz), 5.14 (s, 1H, NH). ^13^C-NMR: δ 23.83, 25.38, 48.46 (Piperidine-C), 29.65, 36.45, 42.02, 43.77 (Adamantane-C), 179.75 (C=S). ESI-MS, *m/z*: 279.3 (M+H)^+^.

**8b**: ^1^H-NMR (CDCl_3_): δ 1.57–1.68 (m, 8H, 6 Adamantane-H & 2 Piperidine-H), 1.89–1.90 (m, 1H, Piperidine-H), 2.10 (s, 3H, Adamantane-H), 2.23–2.24 (m, 6H, Adamantane-H), 2.65–2.71 (m, 2H, Piperidine-H), 2.91–2.97 (m, 2H, Piperidine-H), 3.18–3.22 (m, 2H, Piperidine-H), 5.23 (s, 1H, NH), 7.12–7.16 (m, 3H, Ar-H), 7.22–7.25 (m, 2H, Ar-H). ^13^C-NMR: δ 29.23, 42.71, 48.12 (Piperidine-C), 29.78, 32.88, 36.46, 42.0 (Adamantane-C), 126.55, 126.78, 128.49, 145.04 (Ar-C), 179.99 (C=S). ESI-MS, *m/z*: 355.4 (M+H)^+^.

**8c**: ^1^H-NMR (CDCl_3_): δ 1.18 (t, 3H, CH_2_C***H***_3_, *J* = 7.0 Hz), 1.50–2.21 (m, 4H, Piperidine-H), 1.58–1.62 (m, 8H, 6 Adamantane-H & 2 Piperidine-H), 2.04 (s, 3H, Adamantane-H), 2.25–2.27 (m, 6H, Adamantane-H), 2.47–2.58 (m, 1H, Piperidine-H), 3.0–3.14 (m, 2H, Piperidine-H), 4.06 (q, 2H, C***H***_2_CH_3_, *J* = 7.0 Hz), 5.18 (s, 1H, NH). ^13^C-NMR: δ 14.21 (CH_2_***C***H_3_), 29.74, 36.41, 40.60, 41.91 (Adamantane-C), 35.53, 43.76, 46.63 (Piperidine-C), 66.6 (***C***H_2_CH_3_), 174.17 (C=O), 180.14 (C=S).

**9**: ^1^H-NMR (CDCl_3_): δ 1.60–1.67 (m, 6H, Adamantane-H), 2.05 (s, 3H, Adamantane-H), 2.25–2.26 (m, 6H, Adamantane-H), 2.85 (t, 2H, *J* = 6.0 Hz, tetrahydroisoquinoline-CH_2_), 3.81 (t, 2H, *J* = 6.0 Hz, tetrahydroisoquinoline-CH_2_), 4.80 (s, 2H, tetrahydroisoquinoline-CH_2_), 5.15 (s, 1H, NH), 7.08–7.15 (m, 4H, Ar-H). ^13^C-NMR: δ 29.68, 32.88, 35.56, 42.01 (Adamantane-C), 29.24, 48.12, 54.82, 126.49, 127.08, 128.33, 129.14, 133.43, 135.50 (tetrahydroisoquinoline-C), 179.46 (C=S). ESI-MS, *m/z*: 327.4 (M+H)^+^.

### 3.3. Synthesis of N,N'-Bis(1-adamantyl)piperazine-1,4-dicarbothioamide **10a** and trans-N,N'-Bis(1-adamantyl)-2,5-dimethylpiperazine-1,4-dicarbothioamide **10b**

A mixture of 1-adamantyl isothiocyanate **4** (774 mg, 4 mmol) and anhydrous piperazine or *trans*-2,5-dimethylpiperazine (2.0 mmol), in ethanol (20 mL), was heated under reflux for 2 h. On cooling, the precipitated crude product were filtered, washed with cold ethanol, dried, and crystallized from DMF.

**10a**: ^1^H-NMR (CDCl_3_): δ 1.25–1.45 (m, 12H, Adamantane-H), 1.49–1.66 (m, 6H, Adamantane-H), 1.87–2.09 (m, 12H, Adamantane-H), 3.08 (s, 8H, Piperazine-H), 9.81 (s, 2H, NH). ^13^C-NMR: δ 27.98, 35.44, 40.08, 41.98 (Adamantane-C), 51.38, 52.08 (Piperazine-C), 179.98 (C=S). ESI-MS, *m/z*: 471.4 (M−H, 100)^−^.

**10b**: ^1^H-NMR (CDCl_3_): δ 0.86 (d, 6H, CH_3_, *J* = 5.0 Hz), 1.08–1.23 (m, 12H, Adamantane-H), 1.46–1.47 (m, 6H, Adamantane-H), 1.86–2.09 (m, 12H, Adamantane-H), 2.25–2.66 (m, 2H, Piperazine H), 3.43–3.54 (m, 2H, Piperazine-H), 3.70–3.76 (m, 2H, Piperazine-H), 9.83 (s, 2H, NH). ^13^C-NMR: δ 14.15 (CH_3_), 28.14, 35.41, 41.38, 42.70 (Adamantane-C), 50.32, 57.41 (Piperazine-C), 180.01 (C=S). ESI-MS, *m/z*: 499.5 (M−H, 100)^−^.

### 3.4. Synthesis of 1-(1-Adamantylthiocarbamoyl)piperidine-4-carbohydrazide **11** and 4-(1-Adamanty)-3-thiosemicarbazide **12**

Hydrazine hydrate (98%, 10 mL) was added to a hot solution of compound **8c** (3.5 g, 0.01 mol) in ethanol (20 mL) and the mixture was heated under reflux with stirring for 20 min. On cooling, the precipitated crude product was filtered, washed with cold ethanol, dried and crystallized from ethanol to yield 2.42 g (72%) of compound **11**. The filtrate was evaporated under reduced pressure and the residue was crystallized from water to yield 0.4 gm (18%) of compound **12**.

**11**: Mp. 216–217 °C. ^1^H-NMR (DMSO-*d_6_*): δ 1.55–1.96 (m, 10H, 6 Adamantane-H & 4 Piperidine-H), 2.06–2.18 (m, 9H, Adamantane-H), 2.32 (m, 1H, Piperidine-H), 2.68–2.92 (m, 4H, Piperidine-H), 5.50 (d, 2H, NH_2_, *J* = 9.5 Hz), 8.08 (t, 1H, NH, *J* = 9.5 Hz), 8.36 (s, 1H, NH). ^13^C-NMR: δ 28.0, 36.05, 40.75, 44.95 (Adamantane-C), 30.85, 41.35, 48.0 (Piperidine-C), 172.80 (C=O), 181.88 (C=S). ESI-MS, *m/z*: 337.3 (M+H, 100)^+^.

**12**: Mp. 195–197 °C. ^1^H-NMR (DMSO-*d_6_*): δ 1.64–2.02 (m, 6H, Adamantane-H), 2.12–2.18 (m, 9H, Adamantane-H), 4.50 (d, 2H, NH_2_, *J* = 10.5 Hz), 7.42 (t, 1H, NH, *J* = 10.5 Hz), 8.36 (s, 1H, NH). ^13^C-NMR: δ 28.99, 35.95, 39.85, 51.91 (Adamantane-C), 179.08 (C=S). ESI-MS, *m/z*: 226.2 (M+H, 100)^+^.

### 3.5. Synthesis of N-(1-Adamantyl)piperidine-4-(5-mercapto-4-phenyl-1,2,4-triazol-3-yl)-1-carbothioamide **14a**,**b**

A mixture of 1-(1-adamantylthiocarbamoyl)piperidine-4-carbohydrazide **11** (673 mg, 2 mmol), methyl or phenyl isothiocyanate (2 mmol), in ethanol (10 mL), was heated under reflux for 6 h. The solvent was then distilled off *in vacuo* to yield the crude products **13a**,**b** which were used in the second step without further purification.

Aqueous sodium hydroxide solution (10%, 10 mL) was added to the crude product **13a** or **13b** and the mixture was heated under reflux for 2 h, then filtered hot. The filtrate was acidified with 37% HCl to pH 1–2 and the precipitated crude products **14a**,**b** were filtered, washed with water and crystallized.

**14a**: ^1^H-NMR (DMSO-*d_6_*): δ 1.67–1.69 (m, 9H, Adamantane-H), 1.92–2.16 (m, 10H, 6 Adamantane-H & 4 Piperidine-H), 2.22–2.24 (m, 1H, Piperidine-H), 2.62–2.64 (m, 4H, Piperidine-H), 3.42 (s, 3H, CH_3_), 8.30 (s, 1H, NH), 9.88 (s, 1H, SH). ^13^C-NMR: δ 22.50 (CH_3_), 28.92, 36.08, 39.88, 41.90 (Adamantane-C), 27.80, 35.05, 51.30 (Piperidine-C), 163.52, 179.95 (Triazole C-3 & C-5), 180.23 (C=S). ESI-MS, *m/z*: 390.4 (M−H, 100)^−^.

**14b**: ^1^H-NMR (DMSO-*d_6_*): δ 1.64–1.65 (m, 9H, Adamantane-H), 2.01–2.06 (m, 6H, Adamantane-H), 2.16–2.22 (m, 5H, Piperidine-H), 2.51–2.52 (m, 4H, Piperidine-H), 7.14–7.51 (m, 5H, Ar-H), 8.32 (s, 1H, NH), 9.63 (s, 1H, SH). ^13^C-NMR: δ 28.94, 35.88, 39.87, 40.03 (Adamantane-C), 28.98, 35.95, 53.31 (Piperidine-C), 124.80, 124.86, 128.18, 139.0 (Ar-C), 161.50, 180.50 (Triazole C-3 & C-5), 181.90 (C=S). ESI-MS, *m/z*: 452.4 (M−H, 100)^−^.

### 3.6. Synthesis of 1-[(1-Adamantyl)thiocarbamoyl)]-N'-(arylidene)piperidine-4-carbohydrazides **15a**–**g**

A mixture of 1-(1-adamantylthiocarbamoyl)piperidine-4-carbohydrazide **11** (673 mg, 2 mmol), the appropriate aromatic aldehyde (2 mmol), in DMF (6 mL), was heated under reflux for 2 h. On cooling, water (10 mL) was gradually added with stirring and the mixture was allowed to stand for 1 h. The precipitated crude products were filtered, washed with water, dried and crystallized.

**15a**: ^1^H-NMR (DMSO-*d_6_*): δ 1.65–1.67 (m, 9H, Adamantane-H), 1.95–2.40 (m, 15H, 6 Adamantane-H & 9 Piperidine-H), 6.83–6.89 (m, 2H, Ar-H), 7.21–7.23 (m, 1H, Ar-H), 7.47 (s, 1H, NH), 7.68–7.70 (m, 1H, Ar-H), 8.32 (s, 1H, NH), 8.39 (s, 1H, OH), 11.27 (s, 1H, CH=N). ^13^C-NMR: δ 29.01, 35.91, 39.03, 40.98 (Adamantane-C), 30.63, 40.03, 52.92 (Piperidine-C), 116.12, 119.35, 120.29, 125.84, 131.07, 156.51 (Ar-C), 138.50 (CH=N), 162.0 (C=O), 174.54 (C=S). ESI-MS, *m/z*: 439.4 (M−H, 100)^−^.

**15b**: ^1^H-NMR (CDCl_3_): δ 1.65–1.67 (m, 9H, Adamantane-H), 1.91–2.26 (m, 11H, 6 Adamantane-H & 5 Piperidine-H), 2.82–2.89 (m, 4H, Piperidine-H), 7.19–7.66 (m, 4H, Ar-H & NH), 8.48 (s, 1H, NH), 9.29 (s, 1H, CH=N). ^13^C-NMR: δ 29.23, 36.33, 40.67, 41.48 (Adamantane-C), 29.64, 43.78, 54.47 (Piperidine-C), 126.18, 127.70, 128.50, 130.87, 133.33, 133.52 (Ar-C), 138.35 (CH=N), 160.26 (C=O), 174.81 (C=S). ESI-MS, *m/z* (Rel. Int.): 498.4 (M+4+H, 9)^+^, 496.4 (M+2+H, 54)^+^, 494.40 (M+H, 100)^+^.

**15c**: ^1^H-NMR (DMSO-*d_6_*): δ 1.64–1.66 (m, 9H, Adamantane-H), 2.05–3.31 (m, 15H, 6 Adamantane-H & 9 Piperidine-H), 7.40 (t, 1H, Ar-H, *J* = 8.0 Hz), 7.56 (d, 2H, Ar-H, *J* = 8.0 Hz), 7.47 (s, 1H, NH), 7.68–7.70 (m, 1H, Ar-H), 8.34 (s, 1H, NH), 8.39 (s, 1H, CH=N). ^13^C-NMR: δ 28.99, 35.96, 39.04, 41.38 (Adamantane-C), 28.94, 40.86, 52.96 (Piperidine-C), 129.05, 129.70, 130.80, 133.68 (Ar-C), 135.65 (CH=N), 161.50 (C=O), 174.94 (C=S). ESI-MS, *m/z* (Rel. Int.): 498.4 (M+4+H, 11)^+^, 496.4 (M+2+H, 61)^+^, 494.40 (M+H, 100)^+^.

**15d**: ^1^H-NMR (CDCl_3_): δ 1.58–1.72 (m, 9H, Adamantane-H), 1.90–2.27 (m, 11H, 6 Adamantane-H & 5 Piperidine-H), 2.81–2.88 (m, 4H, Piperidine-H), 3.14 (s, 1H, NH), 3.86 (s, 3H, OCH_3_), 3.91 (s, 3H, OCH_3_), 6.84 (d, 1H, Ar-H, *J* = 8.5 Hz), 7.18–7.20 (m, 1H, Ar-H), 7.48 (s, 1H, Ar-H), 8.54 (s, 1H, NH), 8.85 (s, 1H, CH=N). ^13^C-NMR: δ 28.96, 35.54, 41.25, 41.53 (Adamantane-C), 29.74, 43.77, 54.97 (Piperidine-C), 55.99 (OCH_3_), 56.0 (OCH_3_), 108.83, 110.70, 124.05, 127.20, 149.42, 151.91 (Ar-C), 138.55 (CH=N), 161.21 (C=O), 174.81 (C=S). ESI-MS, *m/z*: 485.4 (M+H, 100)^+^.

**15e**: ^1^H-NMR (CDCl_3_): δ 1.63–1.67 (m, 9H, Adamantane-H), 1.90–2.27 (m, 11H, 6 Adamantane-H & 5 Piperidine-H), 2.80–2.86 (m, 4H, Piperidine-H), 3.82 (s, 3H, OCH_3_), 3.83 (s, 6H, OCH_3_), 3.87 (s, 1H, NH), 6.77 (s, 2H, Ar-H), 8.60 (s, 1H, NH), 9.16 (s, 1H, CH=N). ^13^C-NMR: δ 29.64, 35.54, 36.33, 41.49 (Adamantane-C), 29.22, 43.77, 54.22 (Piperidine-C), 56.20 (2xOCH_3_), 56.30 (OCH_3_), 104.35, 128.79, 141.04, 153.57 (Ar-C), 140.23 (CH=N), 161.67 (C=O), 174.69 (C=S). ESI-MS, *m/z*: 515.4 (M+H, 100)^+^.

**15f**: ^1^H-NMR (CDCl_3_): δ 1.59–2.23 (m, 20H, 15 Adamantane-H & 5 Piperidine-H), 2.81–2.89 (m, 4H, Piperidine-H), 3.78 (s, 3H, OCH_3_), 6.44–6.46 (m, 3H, Ar-H & NH), 7.15–7.19 (s, 2H, Ar-H & NH), 8.52 (s, 1H, OH), 11.27 (s, 1H, CH=N). ^13^C-NMR: δ 29.03, 35.87, 41.24, 42.10 (Adamantane-C), 29.88, 36.07, 54.93 (Piperidine-C), 55.50 (OCH_3_), 101.23, 107.50, 111.12, 122.0, 162.82, 163.88 (Ar-C), 133.50 (CH=N), 161.77 (C=O), 174.50 (C=S). ESI-MS, *m/z*: 469.4 (M−H, 100)^−^.

**15g**: ^1^H-NMR (CDCl_3_): δ 1.40 (t, 3H, CH_2_C***H***_3_, *J* = 7.0 Hz), 1.63–2.26 (m, 20H, 15 Adamantane-H & 5 Piperidine-H), 2.25–2.27 (m, 4H, Piperidine-H), 4.07 (q, 2H, C***H***_2_CH_3_, *J* = 7.0 Hz), 5.5 (br. s, 1H, NH), 6.85–6.87 (m, 1H, Ar-H), 7.03–7.05 (m, 2H, Ar-H), 7.30 (s, 1H, NH), 7.66 (s, 1H, NH), 9.28 (s, 1H, CH=N). ^13^C-NMR: δ 14.84 (CH_2_***C***H_3_), 29.54, 36.34, 40.56, 41.56 (Adamantane-C), 30.64, 41.66, 54.15 (Piperidine-C), 64.65 (***C***H_2_CH_3_), 109.39, 114.68, 121.96, 125.80, 146.17, 148.14 (Ar-C), 141.84 (CH=N), 161.20 (C=O), 174.33 (C=S). ESI-MS, *m/z*: 483.4 (M−H, 100)^+^.

### 3.7. Determination of the in Vitro Antimicrobial Activity (Agar Disc-Diffusion Method)

Sterile filter paper discs (8 mm diameter) were moistened with the compound solution in dimethylsulphoxide of specific concentration (200 μg/disc), the antibacterial antibiotics Gentamicin and Ampicillin trihydrate (100 μg/disc) and the antifungal drug Clotrimazole (100 μg/disc) were carefully placed on the agar culture plates that had been previously inoculated separately with the microorganisms. The plates were incubated at 37 °C, and the diameter of the growth inhibition zones were measured after 24 h in case of bacteria and 48 h in case of *Candida albicans*.

### 3.8. Determination of the Minimal Inhibitory Concentration (MIC)

Compounds **5c**, **5d**, **5e**, **6**, **7**, **10a**, **10b**, **15f** and **15g** Gentamicin and Ampicillin trihydrate were dissolved in dimethylsulphoxide at concentration of 128 μg/mL. The twofold dilutions of the solution were prepared (128, 64, 32, …, 0.5 μg/mL). The microorganism suspensions at 106 CFU/mL (colony forming unit/mL) concentrations were inoculated to the corresponding wells. The plates were incubated at 36 °C for 24 h. The MIC values were determined as the lowest concentration that completely inhibited visible growth of the microorganism as detected by unaided eye.

### 3.9. Determination of the in Vivo Hypoglycemic Activity

*Animals*: Locally bred male Sprauge-Dawley rats (250 ± 30 g body weight) were obtained from Abu Rawash, Giza, Egypt. The rats were housed in wire-bottomed cages at 22 ± 2 °C. A standard pellet diet and tap water were supplied *ad libitium*. The animals were acclimatized to these conditions for 15 days before the experiment.

*Induction of experimental diabetes*: Rats were fasted for 16 h before the induction of diabetes with STZ (Sigma Chemical Co., St. Louis, MO, USA). The animals were injected intraperitoneally with 0.22–0.25 mL of a freshly prepared solution STZ (60 mg/mL in 0.01 *M* citrate buffer, pH 4.5) at a final dose of 60 mg/kg body weight. Only rats with serum glucose levels greater than 250 mg/dL were used in experiments.

*Design of the experiment*: Uniform suspensions of the compounds **5c**, **6**, **8b**, **9**, **14a** and **15b** and the oral hypoglycemic drug gliclazide (positive control) in 0.5% (w/v) aqueous carboxymethyl cellulose (CMC) solution were prepared at specific concentration of 10 mg/mL in case of the test compounds and gliclazide. 48 h post STZ injection, the hypoglycemic activity of the compounds **5c**, **6**, **8b**, **9**, **14a** and **15b** was assessed, the diabetic rats were fasted for 16 h and divided into 14 groups each of 5 animals (n = 5) and the serum glucose level was determined for each group and considered as initial fasting serum glucose (C_0_). Group 1, which served as the negative diabetic control group, received only a single oral dose of 0.5% (w/v) aqueous CMC solution (5 mL/kg). Groups 2 was treated with 10 mg/kg gliclazide in 0.5% (w/v) aqueous CMC (positive control). Groups 3–14 were treated with either a single oral dose of the 10 or 20 mg/kg of the test compounds. All treatments were administered by oral gavage. 24 h after treatment, the blood samples were collected and the serum glucose level (C_24_) was determined for each group.

*Determination of serum glucose*: Blood samples from the tail vein were collected, allowed to clot, centrifuged at 2000 r.p.m. for 10 min. The serum was separated and used in the same day for the measurement of serum glucose levels using commercial glucose oxidase (GO) assay kit (Sigma-Aldrich Co., St. Louis, MO, USA). Blood glucose levels were expressed in mg/dL as mean ± SEM. The data were statistically analyzed using ANOVA with Tukey’s multiple comparison test. The values of *p* < 0.01 were considered as significant. The percentage of serum glucose reduction for each group was calculated in relation to the initial serum glucose level as follows:

% Serum glucose reduction = [(C_0_ − C_24_/C_0_)] × 100

where C_0_ is the mean initial fasting serum glucose level, C_24_ is the mean serum glucose level 24 h after treatment.

### 3.10. Determination of the Oral Acute Toxicity of Compound **5c**

Freshly prepared suspensions of compound **5c** in concentrations of 1%, 3%, 4%, 6%, 8% and 12% in 0.5% aqueous carboxymethyl cellulose solution were prepared. Each compound was given to six groups each of 6 normal albino mice of both sexes by oral intubation in doses of 250, 500, 750, 1000, 1250 and 1500 mg/kg. The percentage mortality was recorded 24 h after compound administration and the oral lethal dose LD_50_ was calculated.

## 4. Conclusions

In this study, series of *N*-(1-adamantyl)carbothioamides were synthesized and their *in vitro* antimicrobial activity was determined. Compounds **5c**, **5d**, **5e**, **6**, **7**, **10a**, **10b**, **15a**, **15f** and **15g** displayed marked antibacterial activity. In addition, the *in vivo* oral hypoglycemic activity of compounds **5a**–**e**, **6**, **7**, **8a**–**c**, **10a**, **10b**, **14a**, **14b** and **15a**–**g** was determined in streptozotocin (STZ)-induced diabetic rats. Compound **5c** produced significant hypoglycemic activity compared gliclazide at a safe dose. The active compounds are considered to be good candidates as newer antibacterial and hypoglycemic agents, further studies such as molecular docking for the exploration of the mechanism of their biological activity are required for optimization of the activity are being undertaken.

## Supplementary Materials

Supplementary materials can be accessed at: http://www.mdpi.com/1420-3049/20/05/8125/s1.
