# Supplementary material for: Synthesis, Antimicrobial and Hypoglycemic Activities of Novel N-(1-Adamantyl)carbothioamide Derivatives
Source: Molecules. 2015 May 6;20(5):8125–43. doi: 10.3390/molecules20058125 (PMC6272754; doi:10.3390/molecules20058125)
Supplement: Supplementary file 1 [file molecules-20-08125-s001.pdf]

Supplementary Materials

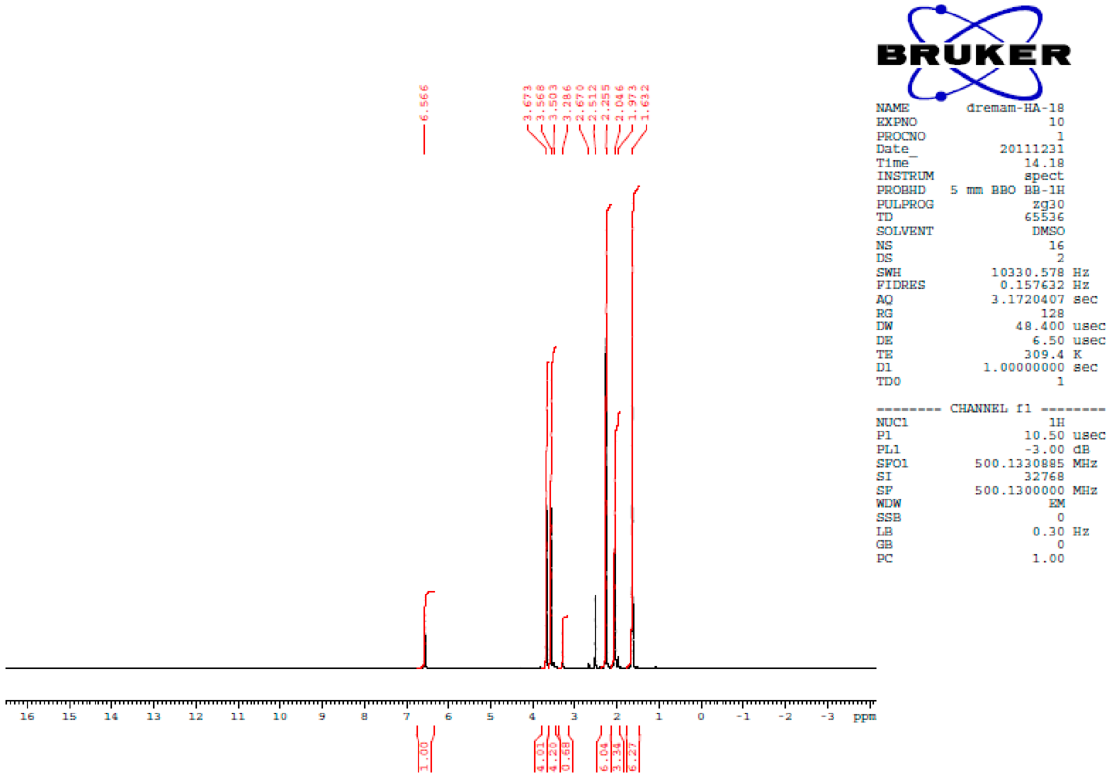

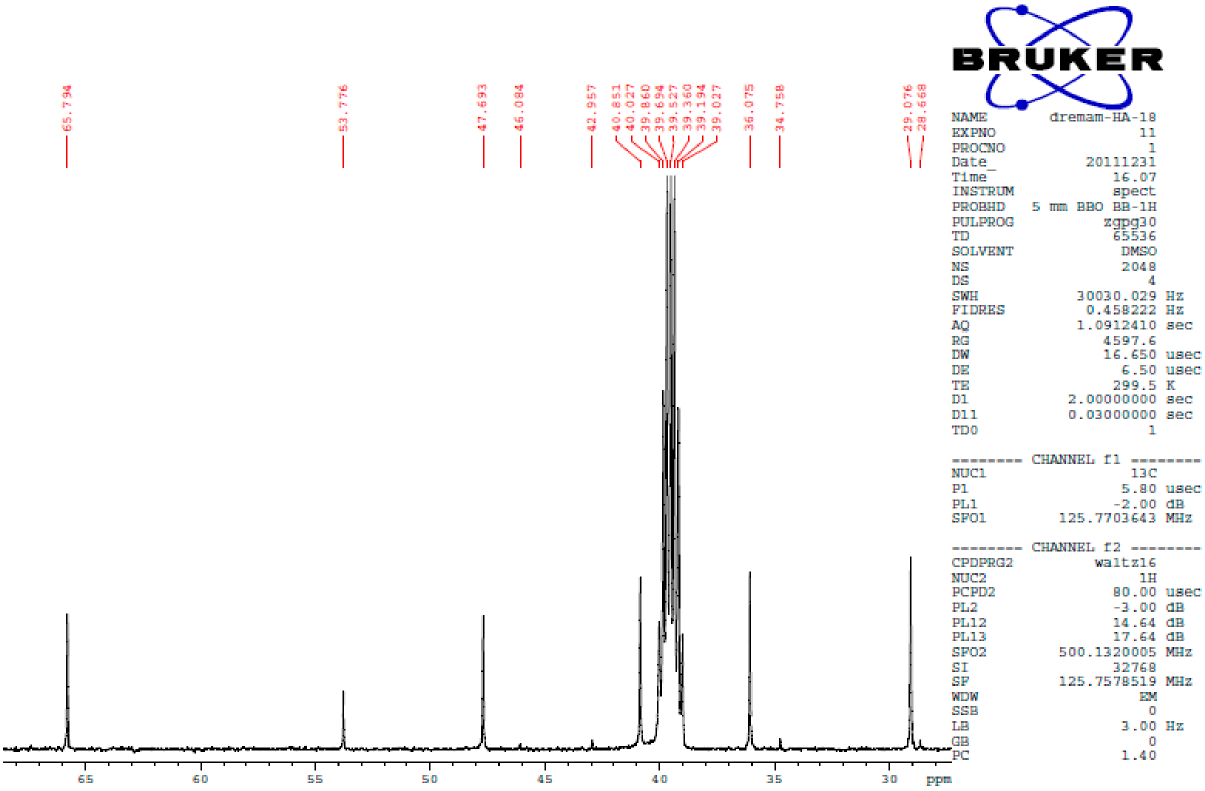

Figure S1. ^{1}H-NMR Spectrum of Compound **6**.

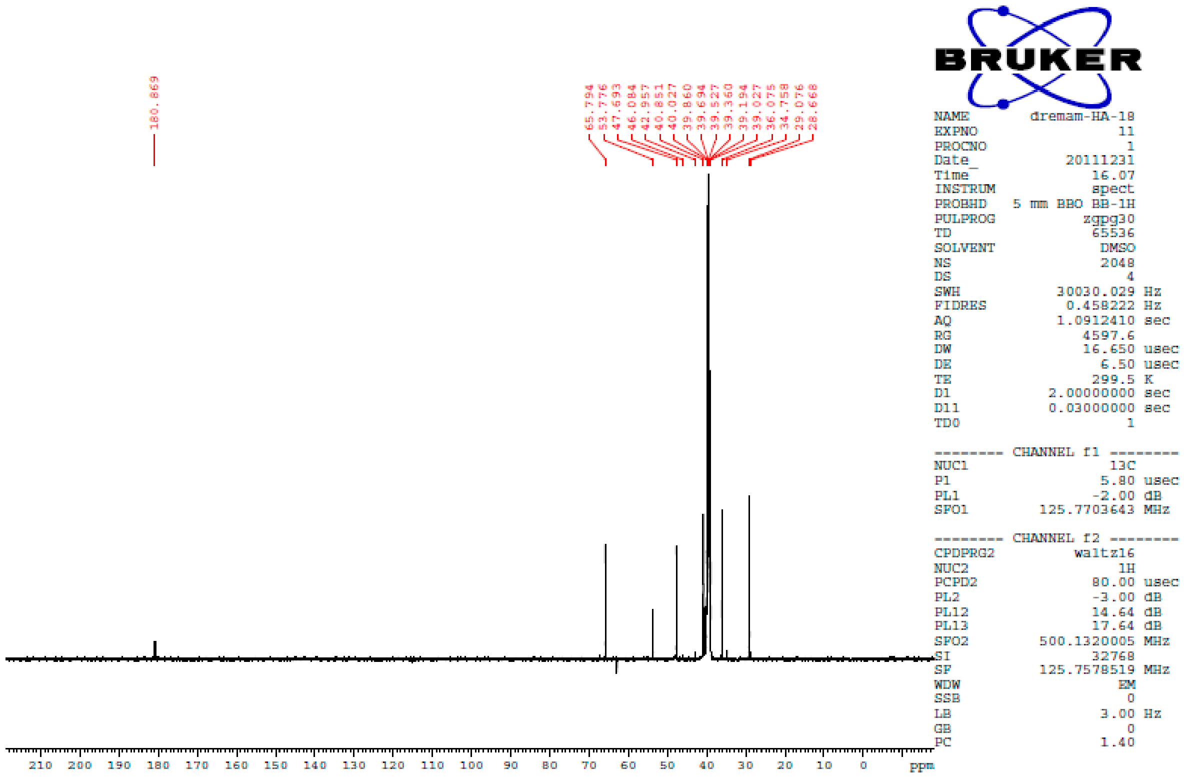

3BO DMSO D:\\ mmj

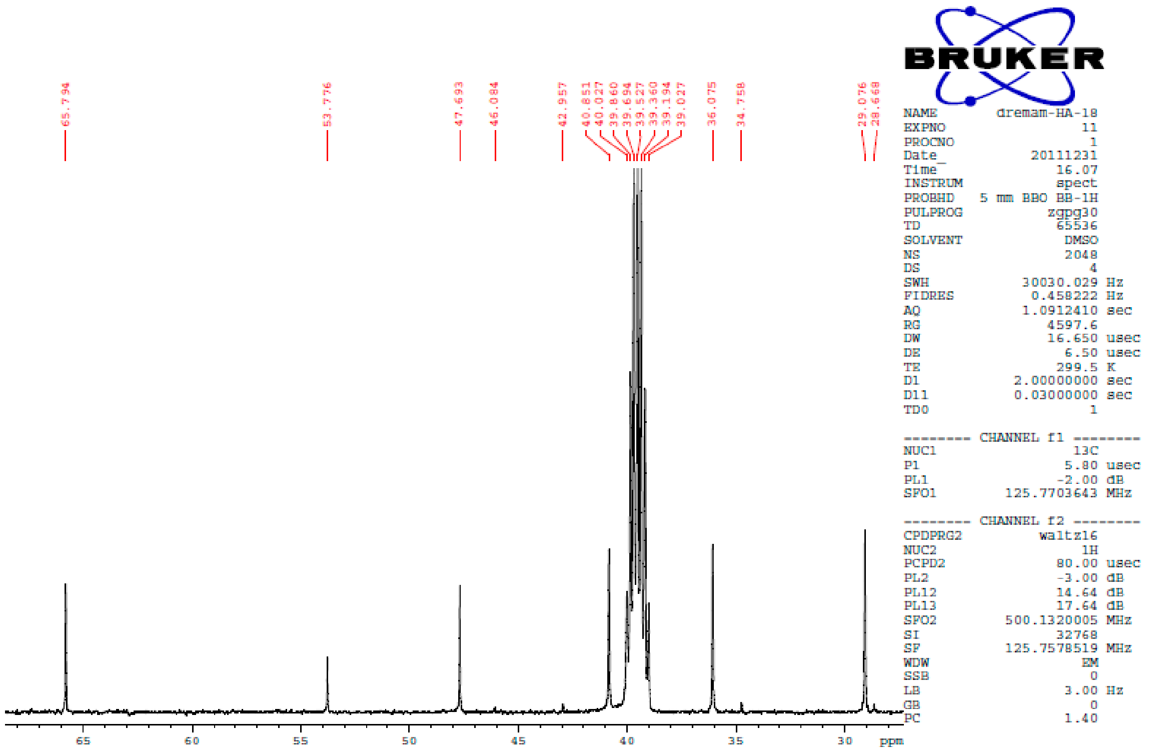

Figure S2. ^{13}C-NMR Spectrum of Compound **6**.

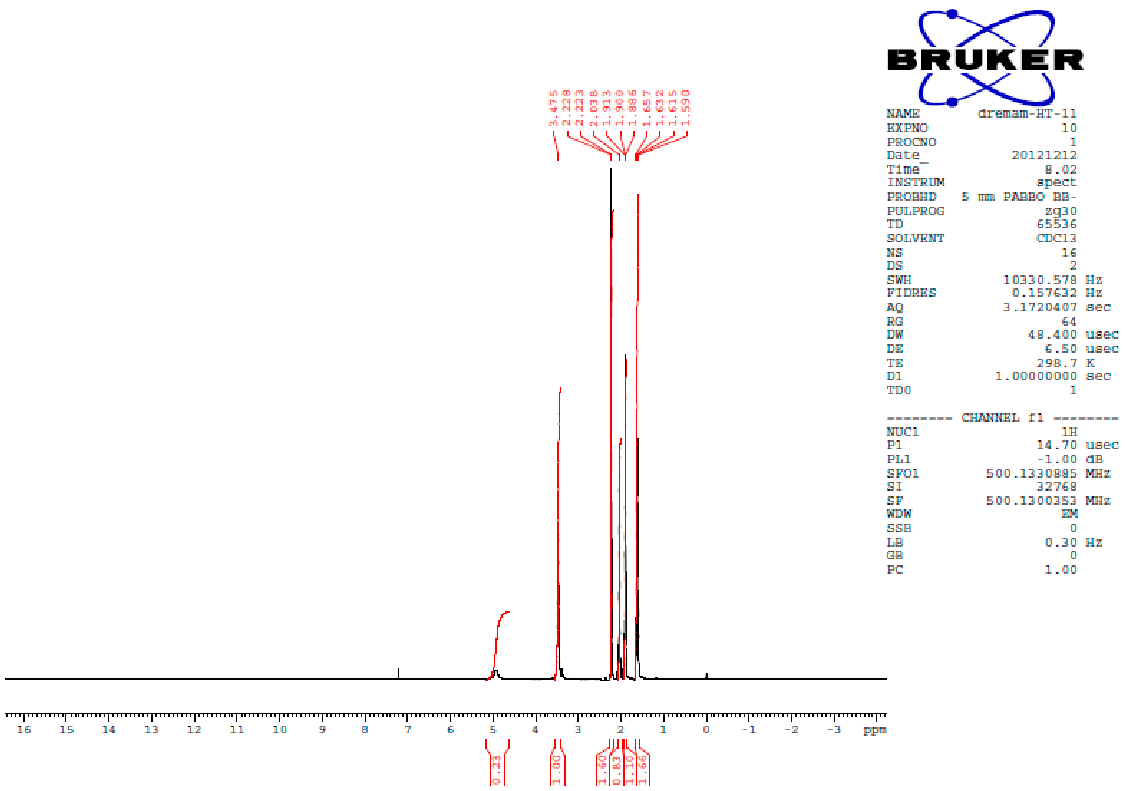

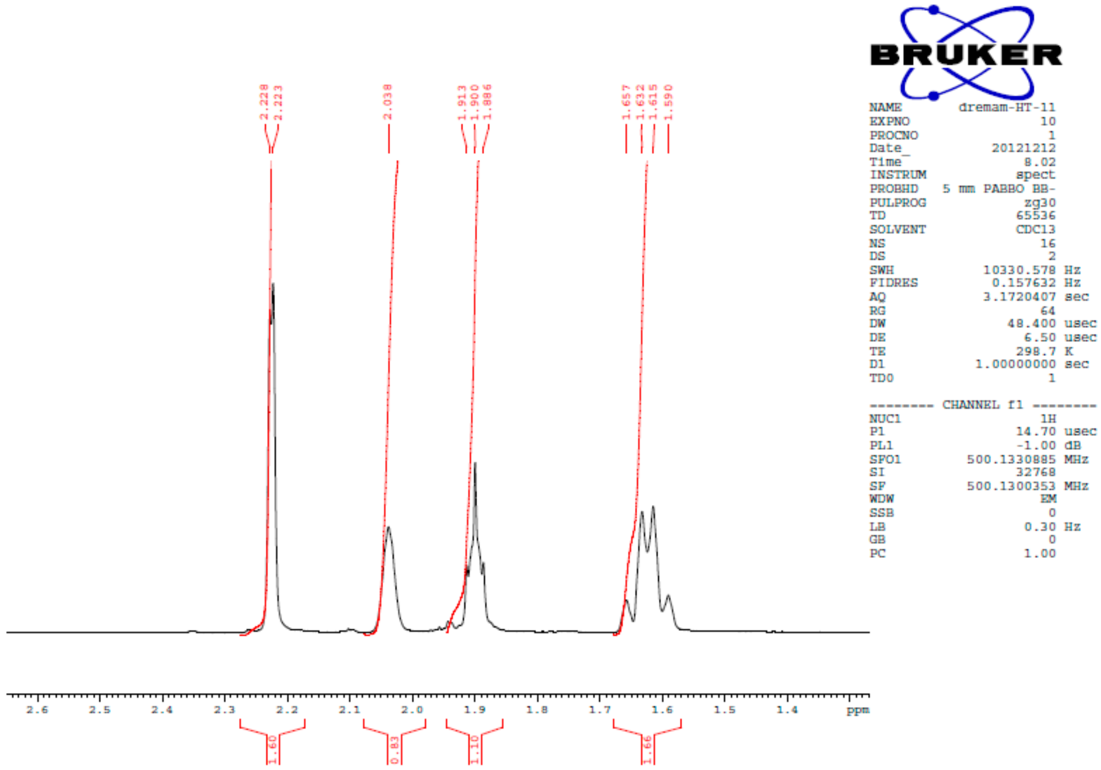

Figure S3. ^{1}H-NMR Spectrum of Compound **7**.

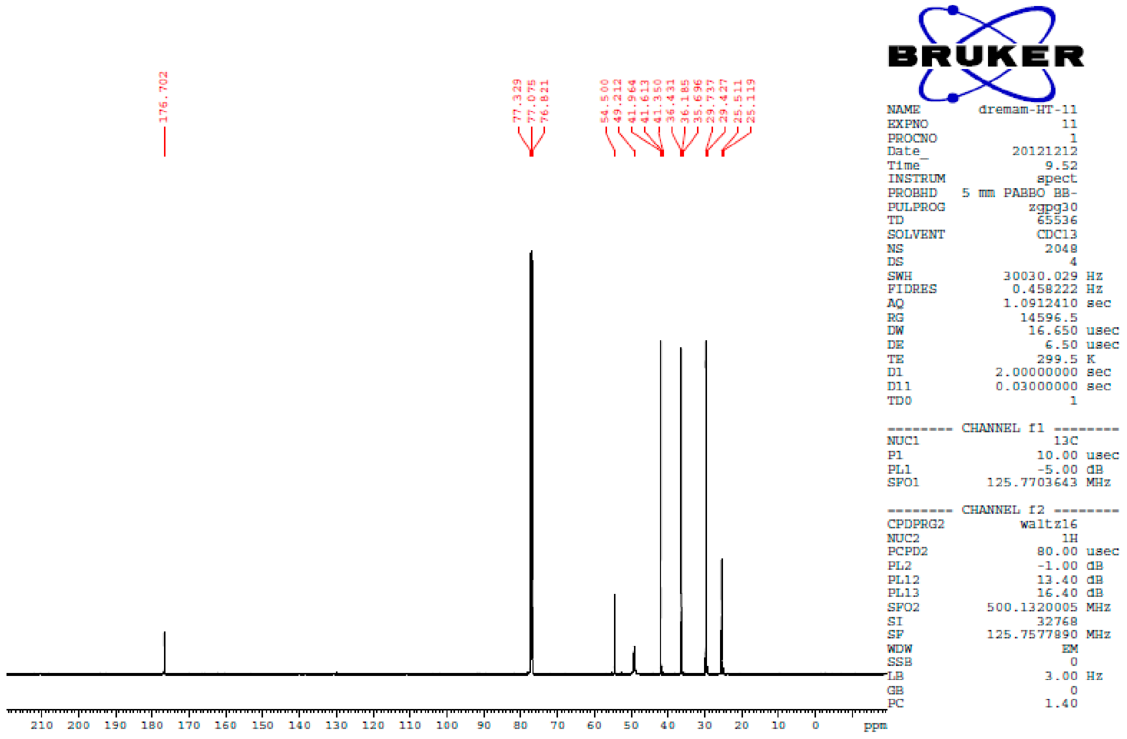

Figure S4. ^{13}C-NMR Spectrum of Compound **7**.

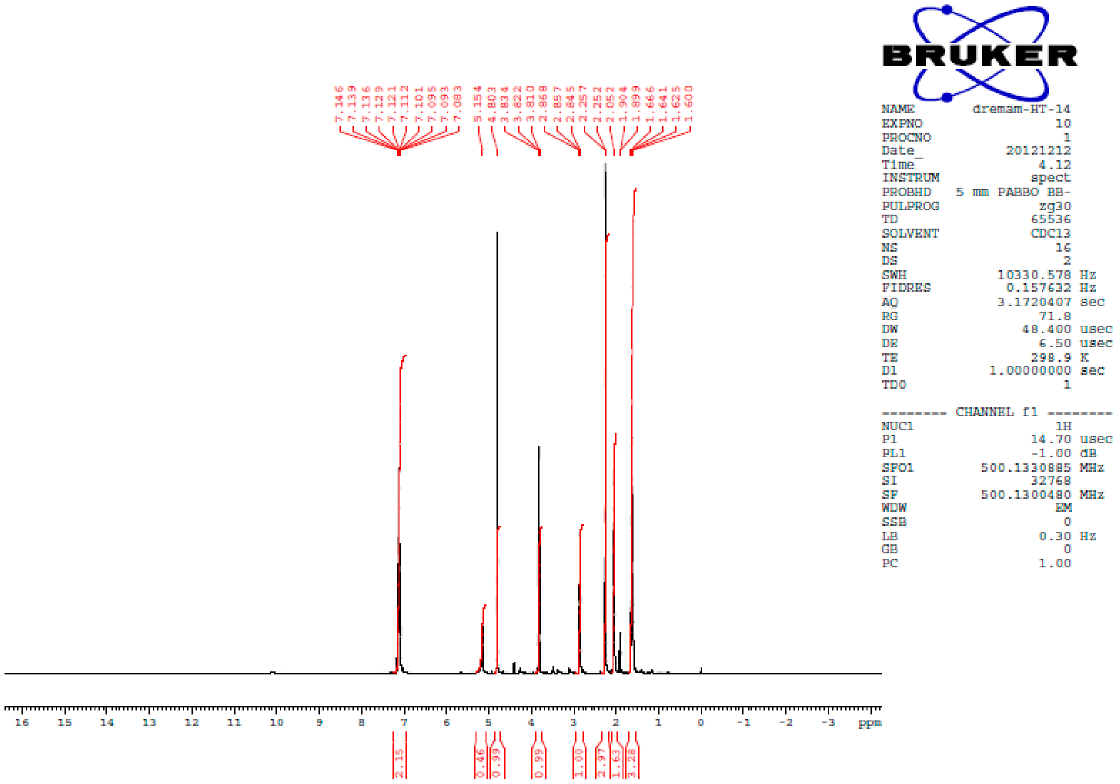

Figure S5. *Cont.*

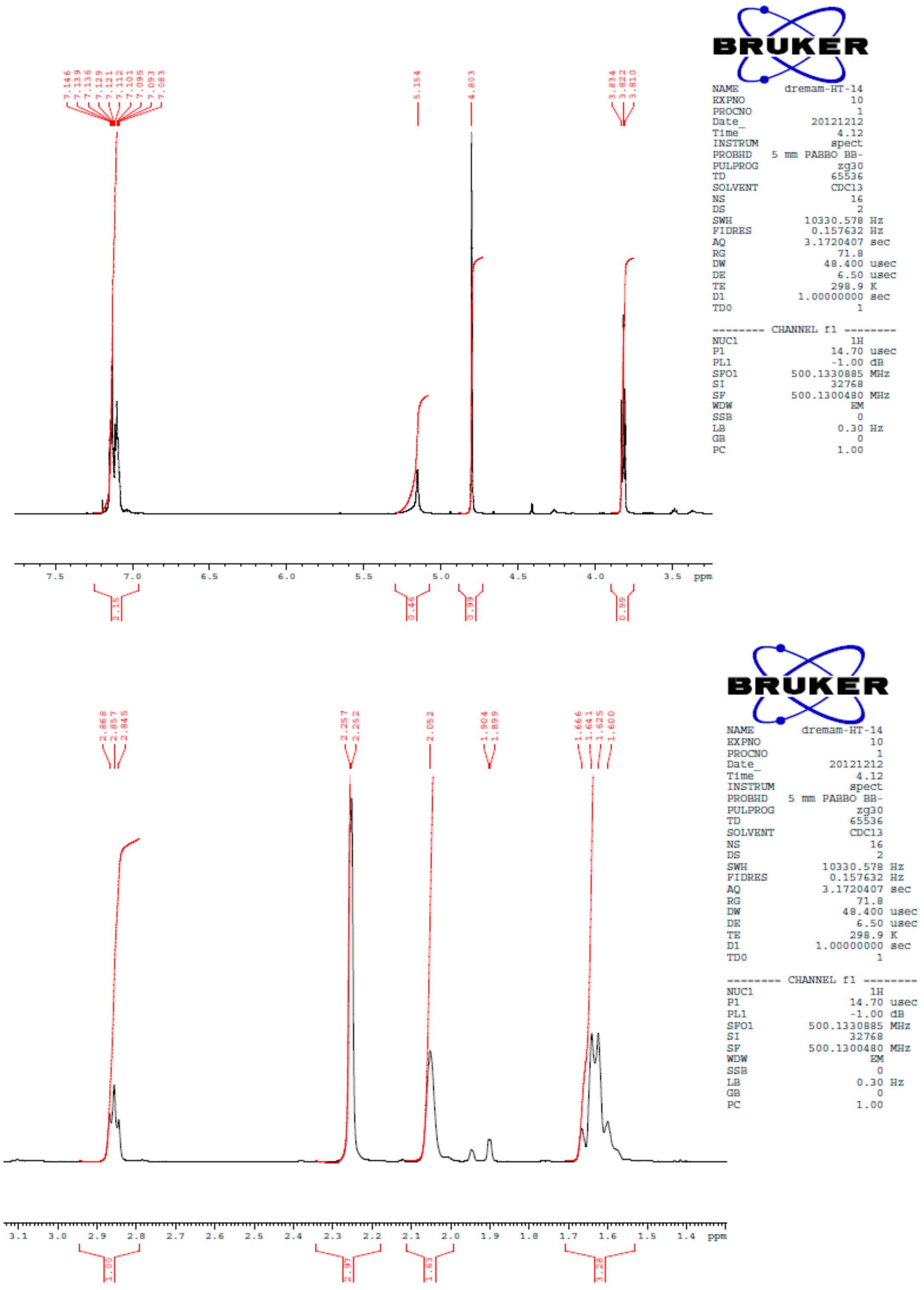

Figure S5. ^{1}H-NMR Spectrum of Compound **9**.

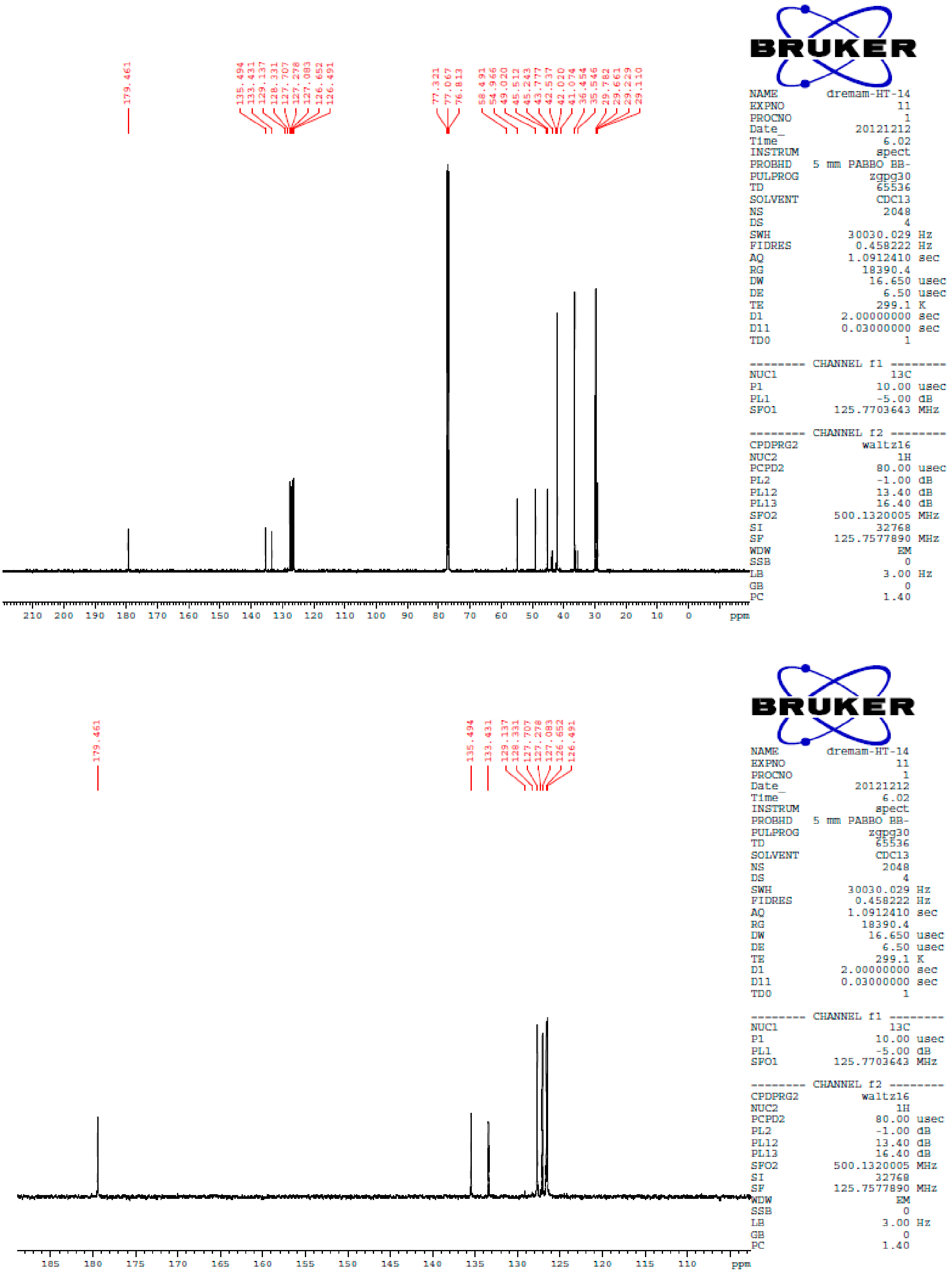

Figure S6. *Cont.*

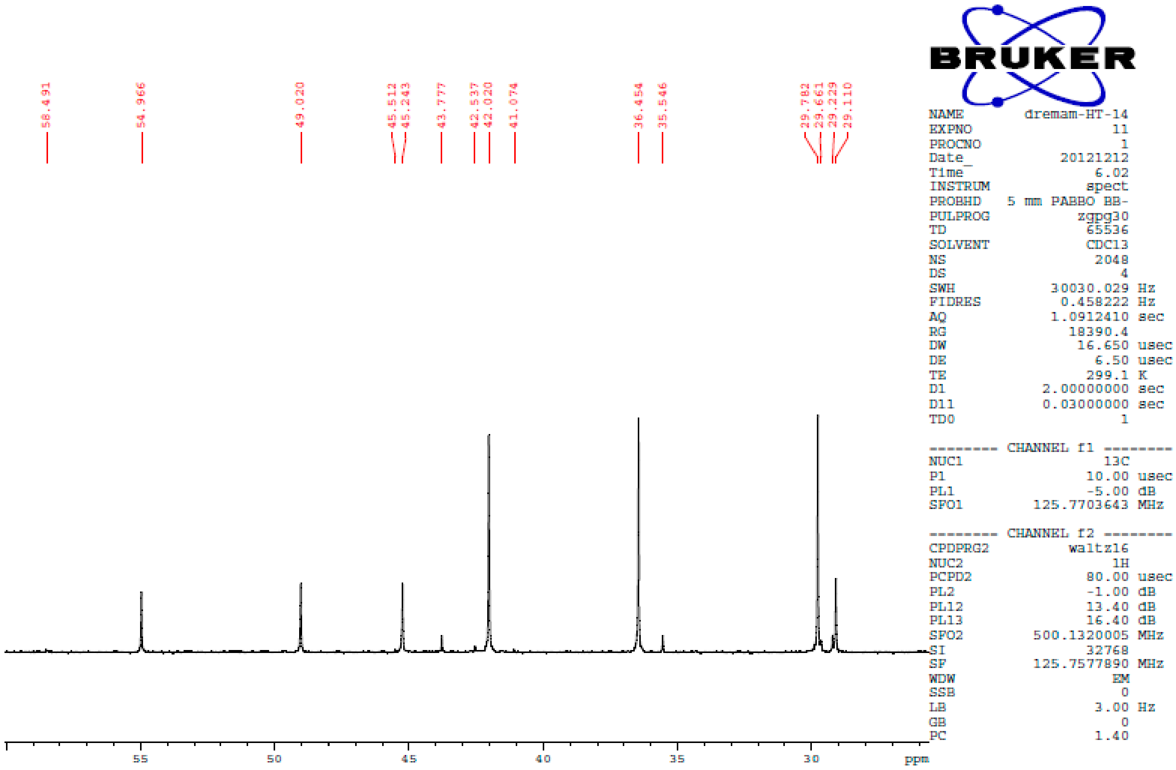

Figure S6. ^{13}C-NMR Spectrum of Compound **9**.
